# Comparison of mortality trends in patients with rheumatic mitral valve disease and nonrheumatic mitral valve disease: A retrospective study in US from 1999 to 2020

**DOI:** 10.1016/j.ijcha.2025.101687

**Published:** 2025-04-24

**Authors:** Eeman Ahmad, Shahzaib Ahmed, Sophia Ahmed, Hamza Ashraf, Umar Akram, Shoaib Ahmad, Irfan Ullah, Mohammed Khanji, Wael Awad, Vuyisile Nkomo, Fabrizio Ricci, Matthew Bates, Mohammad Alkhalil, Raheel Ahmed, Anwar A. Chahal

**Affiliations:** a Department of Medicine, Fatima Memorial Hospital College of Medicine and Dentistry, Lahore, Pakistan; b Department of Medicine, Allama Iqbal Medical College, Lahore, Pakistan; c St. Joseph Hospital and Medical Center, Phoenix, AZ, USA; d Department of Internal Medicine, Khyber Teaching Hospital, Peshawar, Pakistan; e Department of Cardiology, Barts Heart Centre, Barts Health NHS Trust, London, the United Kingdom of Great Britain and Northern Ireland; f Department of Cardiovascular Diseases, Mayo Clinic, Rochester, MN, USA; g Institute for Advanced Biomedical Technologies, “G. d’Annunzio” University of Chieti-Pescara, Via dei Vestini 33 - 66100 Chieti, Italy; h Department of Neuroscience, Imaging and Clinical Sciences, “G. d’Annunzio” University of Chieti-Pescara, Via dei Vestini 33 - 66100 Chieti, Italy; i University Cardiology Division, SS Annunziata Polyclinic University Hospital, Via dei Vestini 5 - 66100 Chieti, Italy; j James Cook University Hospital, Middlesbrough, the United Kingdom of Great Britain and Northern Ireland; k Department of Cardiology, Freeman Hospital, Newcastle upon Tyne, the United Kingdom of Great Britain and Northern Ireland; l National Heart and Lung Institute, Imperial College London, London, the United Kingdom of Great Britain and Northern Ireland; m Center for Inherited Cardiovascular Diseases, WellSpan Health, Lancaster, PA, USA; n Barts Heart Centre, St Bartholomew’s Hospital, Barts Health NHS Trust, London, West Smithfield, the United Kingdom of Great Britain and Northern Ireland; o NIHR Barts Biomedical Research Centre, William Harvey Research Institute, Queen Mary University of London, London, the United Kingdom of Great Britain and Northern Ireland

**Keywords:** Mitral valve disease, Rheumatic mitral valve disease, Nonrheumatic mitral valve disease, Mortality

## Abstract

Background: Mitral valve disease (MVD) can have both rheumatic and nonrheumatic etiologies. However, differences in mortality remain unknown. Methods: We extracted age-adjusted mortality rates (AAMRs) per 100,000 persons from the CDC WONDER database and stratified them by sex, region, and race. Annual percent change (APC) and average annual percent change (AAPC) were calculated using Joinpoint regression. Pairwise comparison was used to identify significant differences for MVD mortality trends between rheumatic and nonrheumatic patients. Results: From 1999 to 2020, a total of 72,085 deaths were recorded in patients with rheumatic MVD, while 132,300 occurred in those with nonrheumatic MVD. The AAMR for nonrheumatic patients was twice as high as that for rheumatic patients throughout the study period, and AAPC differed significantly between the groups (p < 0.05). Females with rheumatic MVD were observed to have a higher AAMR (1.1) than their male counterparts (0.8), but those with nonrheumatic MVD exhibited a similar AAMR for both females and males. Among rheumatic patients, NH (non-hispanic) Whites were reported to have the highest AAMR (1.0), followed by NH Black or African Americans (0.8), Hispanic or Latinos (0.7), and NH Asian or Pacific Islanders (0.7). Among nonrheumatic patients, NH Whites exhibited the highest AAMR (1.9), followed by NH Black or African Americans (1.4), NH Asian or Pacific Islanders (1.1), and Hispanic or Latinos (1.0). The AAMR in Rural areas was higher than that in Urban areas for patients with both rheumatic and nonrheumatic MVD. Conclusion: Comprehensive public health strategies are essential to improving survival outcomes.

## Graphical abstract

**Figure:**
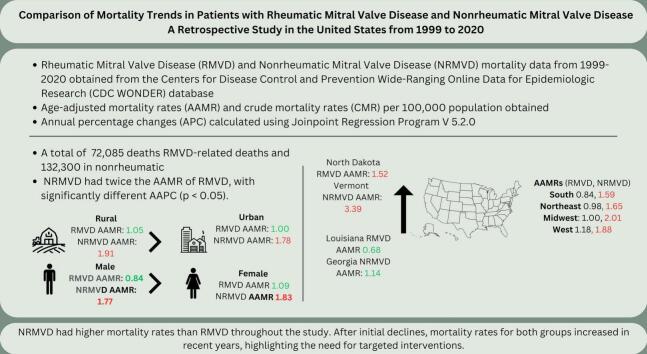

## 1 Introduction

Rheumatic heart disease (RHD), a severe complication of acute rheumatic fever, develops due to an abnormal immune response triggered by Group A streptococcal infections. This immune response leads to progressive valve damage, primarily targeting the mitral valve (MV). Globally, RHD affects over 40.5 million individuals, resulting in more than 300,000 deaths annually [1,2]. It continues to be a significant cause of morbidity and mortality among children and young adults, particularly in developing countries, with the highest prevalence seen in individuals aged 25–35 years [3]. In contrast, RHD is far less prevalent in developed nations, with a reported prevalence of 0.05 per 1,000 individuals in the United States (US). This disparity is attributed to advancements in living standards, access to high-quality healthcare, and widespread availability of antibiotics. However, despite these improvements, an increase in RHD incidence has been observed in developed countries over the past two decades [[4], [5], [6], [7], [8]].

Mitral valve disease (MVD) can also arise from nonrheumatic causes, including obstructive lesions such as large atrial myxomas or infected vegetations, mitral annular calcification, radiation-induced valvulitis, congenital abnormalities, systemic inflammatory disorders such as lupus erythematosus and rheumatoid arthritis [[9], [10], [11], [12]]. Pathological changes at any level of the MV apparatus can lead to functional impairments, which are broadly classified as mitral stenosis, mitral regurgitation, or a combination of both. These conditions often result in hemodynamic disturbances and progressive cardiac dysfunction.

The rising prevalence of MVD, regardless of etiology, coupled with its associated mortality rates, has emerged as a critical challenge for healthcare systems worldwide. In this context, our study aims to provide a comprehensive analysis comparing mortality trends related to rheumatic and nonrheumatic MVD in the US over the past two decades. By examining mortality patterns across various demographic groups and comparing the two different etiologies, we seek to better understand the evolving burden of these conditions and the disparities in their impact on different populations.

## 2 Methods

### 2.1 Study design

We extracted data from the Centers for Disease Control and Prevention Wide‐Ranging Online Data for Epidemiologic Research (CDC WONDER) Database [13] to analyze rheumatic MVD and nonrheumatic MVD-related deaths in the US from 1999 to 2020. CDC WONDER utilizes death certificate data to present both underlying and multiple causes of death as well as demographic information. Annually, the data is updated with information from death certificates of US residents, which includes both cause of death and demographic details. We used the International Classification of Diseases, 10th Revision (ICD‐10) [14] codes I05 (Rheumatic mitral valve diseases) and I34 (Nonrheumatic mitral valve disorders) in the multiple-cause-of-death column to identify death certificates with rheumatic MVD or nonrheumatic MVD listed anywhere on them. Since this study utilized anonymized, publicly available data, it did not require ethical approval from an Institutional Review Board (IRB). This study conformed to the Strengthening the Reporting of Observational Studies in Epidemiology (STROBE) guidelines [15].

### 2.2 Data abstraction

We extracted data and stratified it by sex, race/ethnicity, census region, urbanization, age group, and state. Sex was defined as either male or female. Race/ethnicity groups included NH (non-Hispanic) White, NH Asian or Pacific Islander, NH Black or African American, NH American Indian or Alaska Native, and Hispanic or Latino. Categories for census regions of the US included different census regions including Northeast, Midwest, South and West. County‐level urbanization classifications according to the 2013 National Center for Health Statistics' (NCHS) Urban-Rural Classification Scheme for Counties [16] was used to classify regions as either rural (micropolitan and noncore regions) or urban (large central metro, large fringe metro, medium metro and small metro regions). Age groups were divided into ten-year intervals.

### 2.3 Statistical analysis

Rheumatic MVD and nonrheumatic MVD-related crude mortality rates (CMR) and age-adjusted mortality rates (AAMR) per 100,000 persons were obtained separately. Trends in mortality rates were analyzed using the Joinpoint Regression Program (Joinpoint version 5.1.0, National Cancer Institute), which uses serial permutation tests to examine repeated time trends and identify up to a single inflection point where the rate of change is significantly different [17]. For each time segment, annual percentage change (APC) is obtained along with its corresponding 95 % confidence interval (CI).

We also performed a pairwise comparison using the same program to examine whether there were statistically significant differences in APCs of rheumatic MVD-related mortality trends and nonrheumatic MVD-related mortality trends across various stratifications. A p-value of < 0.05 was considered to be significant.

## 3 Results

**Fig. 1:**
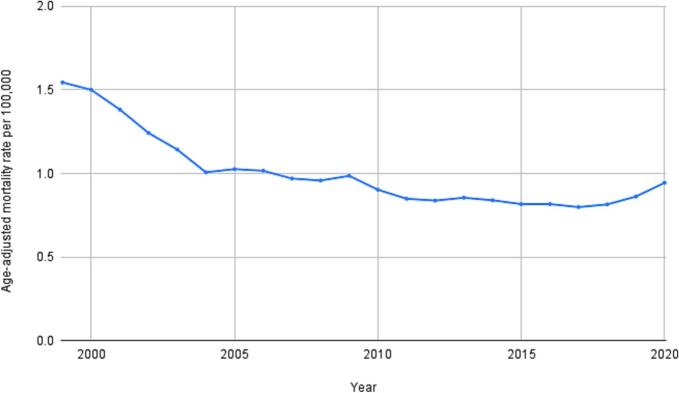
Trends in rheumatic mitral valve disease-related mortality in the United States from 1999 to 2020.

**Fig. 2:**
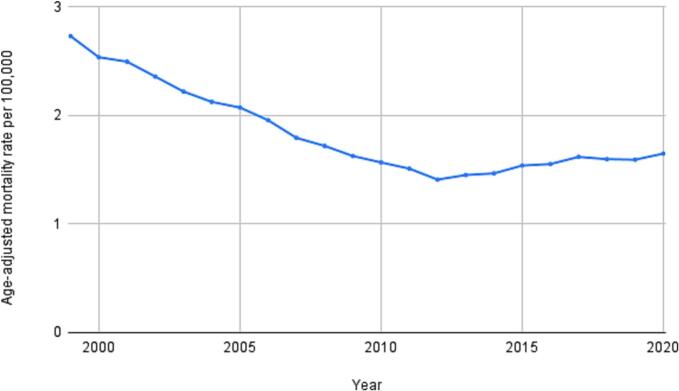
Trends in nonrheumatic mitral valve disease-related mortality in the United States from 1999 to 2020.

From 1999 to 2020, a total of 72,085 deaths were recorded in patients with rheumatic MVD, while 132,300 occurred in those with nonrheumatic MVD (Table 1). The cohort with rheumatic MVD had an average AAMR of 1.8 (95 % CI: 1.79 to 1.81, Table 1), which is approximately twofold higher, compared to nonrheumatic MVD (0.99; 95 % CI: 0.99 to 1.00, Table 1, Fig. 2). Rheumatic MVD mortality rates initially saw a significant decrease from 1999 to 2004 (APC: −8.1*; 95 % CI: −9.7 to −6.6, Table 2, Fig. 1), followed by a more subtle but still significant decline from 2004 to 2017 (APC: −2.2*; 95 % CI: −2.7 to −1.7, Table 2, Fig. 1). This trend then reversed, with a significant increase observed from 2017 to 2020 (APC: 6.0*; 95 % CI: 2.0 to 10.3, Table 2, Fig. 1). Similarly, nonrheumatic MVD mortality rates experienced a significant decline from 1999 to 2012 (APC: −4.9*; 95 % CI: −5.1 to −4.6, Table 2, Fig. 2), followed by a significant rise from 2012 to 2020 (APC: 1.9*; 95 % CI: 1.4 to 2.5, Table 2, Fig. 2). The mortality differed significantly across the groups (p < 0.05, −2.54*; 95 % CI: 3.2 to −1.9 for rheumatic MVD and −2.33*; 95 % CI: −2.6 to −2.1 for nonrheumatic MVD, Table 2).

**Table 1:** Demographic characteristics of deaths rheumatic and nonrheumatic mitral valve disease-related mortality in the United States from 1999 to 2020.

**Variable**	**Population**	**Rheumatic Mitral Valve Disorder**		**Nonrheumatic Mitral Valve Disorder**	
		**Deaths**	**AAMR (95 % CI)**	**Deaths**	**AAMR (95 % CI)**
Overall	6,746,356,647	72,085	0.992 (0.985–––1.000)	132,300	1.8 (1.791–––1.81)
**Sex**					
Female	3,429,003,804	46,133	1.085 (1.075–––1.095)	79,634	1.828 (1.815–––1.841)
Male	3,317,352,843	25,952	0.84 (0.83–––0.851)	52,666	1.765 (1.75–––1.78)
**Age Groups**^a^					
5 to 14 years	901,223,301	61	0.01 (0.01 to 0.01)	105	0.01 (0.01–––0.01)
15 to 24 years	935,794,722	196	0.02 (0.02 to 0.02)	579	0.06 (0.06 to 0.0)
25 to 34 years	920,089,469	641	0.07 (0.06 to 0.08)	1,348	0.15 (0.14 to 0.15)
35 to 44 years	931,287,288	1,720	0.185 (0.176–––0.193)	2,704	0.29 (0.279–––0.301)
45 to 54 years	927,576,220	4,441	0.479 (0.465–––0.493)	5,657	0.61 (0.594–––0.626)
55 to 64 years	766,424,847	8,895	1.161 (1.136–––1.185)	11,020	1.438 (1.411–––1.465)
65 to 74 years	510,458,341	15,253	2.988 (2.941–––3.036)	20,947	4.104 (4.048–––4.159)
75 to 84 years	298,504,433	23,057	7.724 (7.624–––7.824)	40,168	13.456 (13.325–––13.588)
85 + years	119,513,891	17,649	14.767 (14.549–––14.985)	49,638	41.533 (41.168–––41.899)
**Race / Ethnicity**					
Hispanic or Latino	1,077,280,338	4,036	0.703 (0.68–––0.725)	5,284	0.987 (0.959–––1.014)
NH White	4,394,181,258	59,494	1.025 (1.017–––1.034)	113,343	1.929 (1.918–––1.94)
NH Black or African American	863,931,810	5,953	0.813 (0.792–––0.834)	9,982	1.447 (1.418–––1.477)
NH Asian or Pacific Islander	354,753,001	2,042	0.699 (0.668–––0.731)	2,944	1.109 (1.068–––1.15)
**Census Region**					
Northeast	1,212,994,922	13,387	0.921 (0.905–––0.937)	27,522	1.871 (1.848–––1.893)
Midwest	1,466,121,214	17,499	1.063 (1.047–––1.079)	33,590	2.005 (1.983–––2.026)
South	2,497,818,081	22,640	0.838 (0.827–––0.849)	41,689	1.587 (1.572–––1.603)
West	1,569,422,430	18,559	1.18 (1.163–––1.197)	29,499	1.881 (1.86–––1.903)
**Urbanization**					
Urban	5,739,475,649	58,482	0.97 (0.962–––0.978)	107,746	1.78 (1.769–––1.791)
Rural	1,006,871,652	13,603	1.051 (1.033–––1.069)	24,554	1.907 (1.883–––1.931)

Abbreviations: AAMR, age-adjusted mortality rate; CI, confidence interval; NH, non-Hispanic.

a Crude Mortality Rate is used for analysis instead of Age Adjusted Mortality Rates for Age groups.

**Table 2:** Annual Percentage Changes (APCs) and Average Annual Percentage Changes (AAPCs) in rheumatic and nonrheumatic mitral valve disease-related mortality in the United States from 1999 to 2020.

**Variable**	**Rheumatic Mitral Valve Disorder**					**Nonrheumatic Mitral Valve Disorder**			**P-value for AAPC comparison**
	**Lower Endpoint – Upper Endpoint**	**APC (95 % CI)**	**AAPC (95 % CI)**	**P-value**	**Lower Endpoint – Upper Endpoint**	**APC (95 % CI)**	**AAPC (95 % CI)**	**P-value**	
Entire Cohort	1999 – 2004	−8.14* (−10.21 − −6.66)	−2.54* (−2.85 − −2.27)	<0.000001	1999–––2012	−4.86* (−5.13 − −4.63)	−2.33* (−2.47 − − 2.2)	< 0.000001	0.000222*
	2004 – 2017	−2.22* (−2.75 − −1.72)			2012–––2020	1.91* (1.40–––2.48)			
	2017 – 2020	6.03* (2.77–––11.33)							
**Sex**									
Female	1999–––2001	−5.82* (−8.53 − −3.64)	−2.62* (−2.86 − −2.43)	<0.000001	1999–––2012	−4.86* (−5.30 to −4.53)	−2.50* (−2.71 − −2.32)	< 0.000001	0.000222*
	2001–––2004	−10.20* (−11.16 − −1.95)			2012–––2020	1.45* (0.68 to 2.55)			
	2004–––2013	−2.51* (−3.74 − −1.84)							
	2013–––2018	−0.45 (−2.01–––0.97)							
	2018–––2020	7.07* (3.81–––9.25)							
Male	1999–––2004	−6.69* (−12.13 − −4.23)	−2.10* (−2.63 − −1.61)	<0.000001	1999–––2012	−4.81* (−5.22 − −4.47)	−2.19* (−2.38 − −2.00)	< 0.000001	0.000222*
	2004–––2017	−2.26* (−3.31 − −0.82)			2012–––2020	2.22* (1.48–––3.18)			
	2017–––2020	6.86* (1.58–––13.94)							
**Age Groups**									
25 to 34 years					1999–––2011	−4.07* (−9.89 − −2.17)	−1.11* (−2.26 − −0.02)	0.045191	−
					2011–––2020	2.98 (−0.06–––12.89)			
35 to 44 years	1999–––2014	−5.44* (−7.19 − −4.19)	−1.47* (−2.53 − −0.51)	0.003199	1999–––2015	−4.09* (−5.94 − −3.31)	−2.02* (−3.02 − −1.35)	<0.000001	0.067556
	2014–––2020	9.20* (3.55–––23.41)			2015–––2020	4.92 (−0.82–––19.41)			
45 to 54 years	1999–––2006	−9.11* (−15.37 − −6.02)	−3.17* (−3.97 − −2.34)	<0.000001	1999–––2006	0.83 (−1.28–––8.30)	−0.41 (−1.00–––0.61)	0.321936	0.000889*
	2006–––2020	−0.06 (−1.40–––2.19)			2006–––2011	−6.22* (−12.65 − −2.41)			
					2011–––2020	2.00 (−0.04–––8.70)			
55 to 64 years	1999–––2005	−9.50* (−17.87 − −6.18)	−2.56* (−3.48 − −1.77)	<0.000001	1999–––2012	−4.12* (−5.18 − −3.29)	−1.17* (−1.62 − −0.67)	<0.000001	0.001556*
	2005–––2017	−2.32 (−4.71–––0.28)			2012–––2020	3.82 (2.20–––6.22)			
	2017–––2020	11.84* (2.54–––24.62)							
65 to 74 years	1999–––2004	−9.66* (−14.53 − −5.07)	−3.68* (−4.26 − −3.22)	<0.000001	1999–––2012	−6.38* (−8.18 − −5.29)	−3.26* (−3.91 − −2.65)	<0.000001	0.005778*
	2004–––2011	−4.99 (−11.76–––0.98)			2012 – 2020	2.02 (−0.40–––7.42)			
	2011–––2018	−1.32 (−7.00–––1.28)							
	2018–––2020	8.97* (0.69–––14.63)							
75 to 84 years	1999–––2004	−6.88* (−9.23 − −5.57)	−3.00* (−3.29 − −2.74)	<0.000001	1999 – 2012	−5.63* (−6.40 − −5.32)	−3.12* (−3.33 − −2.94)	<0.000001	0.000889*
	2004–––2016	−2.98* (−3.66 − −2.34)			2012 – 2020	1.11* (0.31–––2.19)			
	2016–––2020	1.99 (−0.16–––6.54)							
85 + years	1999–––2004	−6.28* (−11.92 − −3.28)	−1.45* (−1.90 − −0.92)	<0.000001	1999 – 2012	−3.74* (−4.18 − −3.39)	−1.66* (−1.86 − −1.46)	<0.000001	0.000667*
	2004–––2020	0.12 (−0.43–––0.85)			2012 – 2020	1.81* (1.11–––2.80)			
**Race/Ethnicity**									
Hispanic or Latino	1999–––2012	−4.16* (−9.72 − −2.69)	−2.21* (−3.12 − −1.33)	<0.000001	1999–––2012	−5.02* (−7.00 − −3.77)	−2.41* (−3.09 − −1.69)	<0.000001	0.489111
	2012–––2020	1.05 (−1.47–––10.78)			2012–––2020	1.99 (−0.22–––6.24)			
NH White	1999–––2004	−8.58* (−10.64 − −7.14)	−2.55* (−2.88 − −2.27)	<0.000001	1999–––2012	−4.71* (−4.97 − −4.47)	−2.21* (−2.36 − −2.08)	<0.000001	0.000444*
	2004–––2017	−1.94* (−2.55 − −1.43)			2012–––2020	1.98* (1.39–––2.58)			
	2017–––2020	5.52* (1.99–––10.92)							
NH Black or African American	1999–––2004	−8.08* (−13.80 − −5.35)	−1.75* (−2.32 − −1.21)	<0.000001	1999–––2011	−4.05* (−5.18 − −3.19)	−1.36* (−1.76 − −0.92)	<0.000001	0.014222*
	2004–––2017	−2.19* (−3.17 − −0.87)			2011–––2020	2.36 (1.11–––4.19)			
	2017–––2020	11.91 (5.94–––22.31)							
NH Asian or Pacific Islander	1999–––2018	−4.08* (−7.39 − −2.77)	−1.91* (−3.70 − −0.84)	0.002799	1999–––2012	−3.89* (−10.84 − −2.36)	−2.14* (−3.17 − −1.13)	0.000800	0.180444
	2018––2020	21.38 (−2.10–––34.37)			2012–––2020	0.77 (−1.66–––9.89)			
**Census Region**									
Northeast	1999 – 2004	−11.06* (−16.28 − −8.46)	−3.17* (−3.92 − −2.67)	<0.000001	1999 – 2002	−1.54 (−3.96–––2.75)	−3.08* (−3.40 −-2.78)	<0.000001	0.000222*
	2004–––2018	−2.24* (−3.84 − −1.28)			2002 – 2012	−6.62* (−7.76 − −6.13)			
	2018–––2020	12.03 (0.56–––17.84)			2012 – 2015	4.85* (0.92–––6.90)			
					2015 – 2020	−1.34 (−4.11–––0.07)			
Midwest	1999–––2004	−7.08* (−11.83 − −4.81)	−2.27* (−2.71 − −1.81)	<0.000001	1999 – 2011	−4.31* (−5.13 − −3.69)	−1.77* (−2.09 −-1.46)	<0.000001	0.004444*
	2004–––2014	−2.25* (−4.02 − −0.69)			2011 – 2020	1.72* (0.72–––3.23)			
	2014–––2020	1.89 (−0.00–––7.19)							
South	1999–––2005	−7.63* (−12.23 − −5.78)	−2.80* (−3.33 − −2.41)	<0.000001	1999 – 2013	−5.19* (−5.59 − −4.84)	−2.64* (−2.86 −-2.40)	<0.000001	0.003111*
	2005–––2017	−2.07* (−4.41 − −0.99)			2013 – 2020	2.67* (1.56–––4.03)			
	2017–––2020	4.50 (−0.54–––10.39)							
West	1999–––2004	−7.03* (−12.93 − −4.59)	−2.33* (−2.89 − −1.84)	<0.000001	1999 – 2004	−6.60* (−8.03 − −5.57)	−2.09* (−2.27- −1.91)	<0.000001	0.008889*
	2004–––2017	−2.42* (−3.48 − −1.15)			2004 – 2013	−2.66* (−3.39 − −1.97)			
	2017–––2020	6.47 (1.18–––14.21)			2013 – 2018	3.27 (−2.15–––5.22)			
					2018–––2020	−0.98 (−3.03–––2.07)			
**Urbanization**									
Urban	1999–––2004	−8.46* (−10.04 − −7.28)	−2.77* (−3.01 − −2.54)	<0.000001	1999–––2012	−5.06* (−5.57 − −4.83)	−2.60* (−2.78 − −2.46)	<0.000001	0.000222*
	2004–––2017	−2.42* (−2.82 − −2.01)			2012–––2017	2.46 (−5.23–––4.10)			
	2017–––2020	5.89* (3.17–––10.51)			2017–––2020	−0.00 (−2.03–––1.67)			
Rural	1999–––2003	−7.39* (−13.20 − −3.82)	−1.90* (−2.41 − −1.32)	<0.000001	1999–––2012	−4.23* (−5.15 − −3.56)	−1.77* (−2.17 − −1.40)	<0.000001	0.054889
	2003–––2013	−2.41 (−4.06–––0.95)			2012–––2020	2.34* (0.85–––4.67)			
	2013–––2020	2.15 (−0.01–––8.32)							

Abbreviations: APC, annual percent change; AAPC, average annual percent change; CI, confidence interval; NH, non-Hispanic.

* Indicates that the APC or AAPC is significantly different from zero at the alpha = 0.05 level.

### 3.1 Trends by sex

Females with nonrheumatic MVD had a higher AAMR (1.83; 95 % CI: 1.82 to 1.84, Table 1) compared to females with rheumatic MVD (AAMR: 1.09; 95 % CI: 1.08 to 1.10, Table 1) and their male counterparts (1.77; 95 % CI: 1.75 to 1.78 for nonrheumatic MVD and 0.84; 95 % CI: 0.83 to 0.85 for rheumatic MVD, Table 1). For females with rheumatic MVD, mortality rates significantly declined from 1999 to 2001 (APC: −5.8*; 95 % CI: −10.2 to −1.3, Table 2, Supplementary Fig. 1) and again from 2001 to 2004 (APC: −10.2*; 95 % CI: −14.9 to −5.2, Table 2, Supplementary Fig. 1), with a continued decrease until 2018, after which a significant increase was observed from 2018 to 2020 (APC: 7.1*; 95 % CI: 1.3 to 13.1, Table 2, Supplementary Fig. 1). For females with nonrheumatic MVD, mortality rates significantly declined from 1999 to 2012 (APC: −4.9*; 95 % CI: −5.2 to −4.6, Table 2, Supplementary Fig. 2) but showed a significant increase from 2012 to 2020 (APC: 1.5*; 95 % CI: 0.8 to 2.2, Table 2, Supplementary Fig. 2). For males with rheumatic MVD, mortality rates showed a significant decline from 1999 to 2004 (APC: −6.7*; 95 % CI: −9.2 to −4.1, Table 2, Supplementary Fig. 1), followed by a continued but slower decline from 2004 to 2017 (APC: −2.3*; 95 % CI: −3.0 to −1.5, Table 2, Supplementary Fig. 1). However, this trend reversed, with a significant increase observed from 2017 to 2020 (APC: 6.9*; 95 % CI: 0.6 to 13.5, Table 2, Supplementary Fig. 1). For males with nonrheumatic MVD, mortality rates significantly decreased from 1999 to 2012 (APC: −4.8*; 95 % CI: −5.2 to −4.5, Table 2, Supplementary Fig. 2) but increased significantly from 2012 to 2020 (APC: 2.2*; 95 % CI: 1.5 to 3.0, Table 2, Supplementary Fig. 2). The mortality differed significantly across the rheumatic and nonrheumatic subgroups (p = 0.0002 for both males and females, Table 2).

### 3.2 Trends by race/ethnicity

In the rheumatic MVD cohort, the highest AAMR was observed in NH White individuals (1.03; 95 % CI: 1.02 to 1.03, Table 1), while the lowest was seen in NH Asian or Pacific Islander individuals (0.70; 95 % CI: 0.67 to 0.73, Table 1) and Hispanics or Latino (0.70; 95 % CI: 0.68 to 0.73, Table 1). Similarly, in the nonrheumatic MVD cohort, NH White individuals had the highest AAMR (1.93; 95 % CI: 1.92 to 1.94, Table 1), while the lowest AAMR was observed in Hispanic or Latino individuals (0.99; 95 % CI: 0.96 to 1.01, Table 1). The AAMRs for nonrheumatic MVD were higher than those for rheumatic MVD in all racial groups (Table 1***)***.

The mortality rate for rheumatic MVD in NH Asians or Pacific Islanders significantly decreased from 1999 to 2018 (APC: −4.08*; 95 % CI: −5.07 to −3.08, Table 2, Supplementary Fig. 3). From 2018 to 2020, the AAMR for rheumatic MVD showed a non-significant sharp increase (APC: 21.38; 95 % CI: −7.99 to 60.13, Table 2, Supplementary Fig. 3). The AAMR for nonrheumatic MVD significantly decreased from 1999 to 2012 (APC: −3.89*; 95 % CI: −5.60 to −2.15, Table 2, Supplementary Fig. 4). From 2012 to 2020, the AAMR for nonrheumatic MVD showed a non-significant increase (APC: 0.77; 95 % CI: −2.15 to 3.78, Table 2, Supplementary Fig. 4). Among NH African Americans, rheumatic MVD mortality significantly decreased from 1999 to 2004 (APC: −8.08*; 95 % CI: −11.49 to −4.54, Table 2, Supplementary Fig. 3) and continued to decline, albeit at a slower rate, until 2017. From 2017 to 2020, the AAMR for rheumatic MVD significantly increased (APC: 11.91*; 95 % CI: 3.19 to 21.37, Table 2, Supplementary Fig. 3). For nonrheumatic MVD, the AAMR significantly decreased from 1999 to 2011 (APC: −4.05*; 95 % CI: −5.04 to −3.05, Table 2, Supplementary Fig. 4) but showed a significant increase from 2011 to 2020 (APC: 2.36*; 95 % CI: 0.84 to 3.90, Table 2, Supplementary Fig. 4). The AAMR for rheumatic MVD significantly decreased from 1999 to 2012 (APC: −4.16*; 95 % CI: −5.60 to −2.71, Table 2, Supplementary Fig. 3) and then showed an increase until 2020 (APC: 1.05; 95 % CI: −1.57 to 3.75, Table 2, Supplementary Fig. 3). Similarly, for nonrheumatic MVD, the AAMR significantly decreased from 1999 to 2012 (APC: −5.02*; 95 % CI: −6.31 to −3.72, Table 2, Supplementary Fig. 4) but showed an increase from 2012 to 2020 (APC: 1.99; 95 % CI: −0.40 to 4.44, Table 2, Supplementary Fig. 4). The AAMR for rheumatic MVD in NH Whites significantly decreased from 1999 to 2004 (APC: −8.58*; 95 % CI: −10.03 to −7.11, Table 2, Supplementary Fig. 3). It continued to decline, though at a slower rate, until 2017. However, from 2017 to 2020, the AAMR showed a significant increase (APC: 5.52*; 95 % CI: 1.37 to 9.84, Table 2, Supplementary Fig. 3). For nonrheumatic MVD, the AAMR significantly decreased from 1999 to 2012 (APC: −4.71*; 95 % CI: −4.97 to −4.44, Table 2, Supplementary Fig. 4) but increased from 2012 to 2020 (APC: 1.98*; 95 % CI: 1.36 to 2.60, Table 2, Supplementary Fig. 4). The mortality did not differ significantly across the NH Asians and Hispanic rheumatic and nonrheumatic subgroups (p = 0.18 for NH Asians and 0.49 for Hispanic, Table 2), while it differed significantly for NH whites and NH Black or African American (p = 0.0004 for NH Whites and 0.01 for NH Black or African Americans, Table 2).

### 3.3 Trends by census region

In the nonrheumatic MVD cohort, the highest AAMR was observed in the Midwest (2.005; 95 % CI: 1.983 to 2.026, Table 1), while the lowest was seen in the South (1.587; 95 % CI: 1.572 to 1.603, Table 1). While, in the rheumatic MVD cohort, the highest AAMR was observed in the West (1.18; 95 % CI: 1.163 to 1.197, Table 1), while the lowest was seen in the South (0.838; 95 % CI: 0.827 to 0.849, Table 1). The AAMRs for nonrheumatic MVD were higher than those for rheumatic MVD in all regions (Table 1***)***.

The AAMR for rheumatic MVD in the Northeast declined from 1999 to 2018 but increased from 2018 to 2020 (APC: 12.03; 95 % CI: −4.07 to 30.82, Table 2, Supplementary Fig. 5). For nonrheumatic MVD, the AAMR decreased from 1999 to 2012, rose from 2012 to 2015 (APC: 4.85; 95 % CI: −4.23 to 14.80, Table 2, Supplementary Fig. 6), and remained stable from 2015 to 2020 (APC: −1.34; 95 % CI: −3.27 to 0.63, Table 2, Supplementary Fig. 6). The AAMR for rheumatic MVD in the Midwest significantly decreased from 1999 to 2004 (APC: −7.08; 95 % CI: −9.69 to −4.39, Table 2, Supplementary Fig. 5) and from 2004 to 2014 (APC: −2.25; 95 % CI: −3.50 to −0.97, Table 2, Supplementary Fig. 5) but increased from 2014 to 2020 (APC: 1.89; 95 % CI: −0.43 to 4.26, Table 2, Supplementary Fig. 5). For nonrheumatic MVD, the AAMR significantly declined from 1999 to 2011 (APC: −4.31; 95 % CI: −4.80 to −3.82, Table 2, Supplementary Fig. 6) but rose from 2011 to 2020 (APC: 1.72; 95 % CI: 0.90 to 2.55, Table 2, Supplementary Fig. 6). The AAMR for rheumatic MVD in the South region significantly decreased from 1999 to 2017 (Supplementary Fig. 5). However, it increased from 2017 to 2020 (APC: 4.50; 95 % CI: −1.16 to 10.48, Table 2, Supplementary Fig. 5). For nonrheumatic MVD, the AAMR significantly decreased from 1999 to 2013 (APC: −5.19*; 95 % CI: −5.59 to −4.79, Table 2, Supplementary Fig. 6) but significantly increased from 2013 to 2020 (APC: 2.67*; 95 % CI: 1.41 to 3.94, Table 2, Supplementary Fig. 6). The AAMR for rheumatic MVD in the West region significantly decreased from 1999 to 2017 (Supplementary Fig. 5). However, it increased from 2017 to 2020 (APC: 6.47; 95 % CI: −1.07 to 14.58, Table 2, Supplementary Fig. 5). For nonrheumatic MVD, the AAMR decreased from 1999 to 2013 and significantly increased from 2013 to 2018 (APC: 3.27*; 95 % CI: 1.50 to 5.07, Table 2, Supplementary Fig. 6). However, it remained stable from 2018 to 2020 (APC: −0.98; 95 % CI: −5.80 to 4.09, Table 2, Supplementary Fig. 6). The mortality rates differed significantly across the rheumatic and nonrheumatic subgroups (p = 0.0002 for Northeast, 0.004 for Midwest, 0.003 for South, and 0.009 for West, Table 2).

### 3.4 Trends by urbanization

The AAMR for rheumatic MVD was lower in urban areas (1.00; 95 % CI: 0.96 to 0.98, Table 1) compared to rural areas (1.05; 95 % CI: 1.03 to 1.07, Table 1). Similarly, the AAMR for nonrheumatic MVD was lower in urban areas (1.78; 95 % CI: 1.77 to 1.79, Table 1) compared to rural areas (1.91; 95 % CI: 1.88 to 1.93, Table 1). The AAMR for rheumatic MVD in the Urban region significantly decreased from 1999 to 2017 (Table 1, Supplementary Fig. 7***)***. However, it increased from 2017 to 2020 (APC: 5.89*; 95 % CI: 2.06 to 9.86, Table 2, Supplementary Fig. 7). For nonrheumatic MVD, the AAMR significantly decreased from 1999 to 2012 (APC: −5.06*; 95 % CI: −5.28 to −4.85, Table 2, Supplementary Fig. 8) while increased from 2012 to 2017 (APC: 2.46*; 95 % CI: 0.99 to 3.95, Table 2, Supplementary Fig. 8). The AAMR remained stable from 2017 to 2020 (APC: −0.003; 95 % CI: −2.12 to 2.16, Table 2, Supplementary Fig. 8). The mortality did not differ significantly across the rural rheumatic and nonrheumatic subgroups, but differed significantly for urban subgroups (p = 0.0002 for urban and 0.055 for rural, Table 2).

### 3.5 Trends by age group

The crude death rate for nonrheumatic MVD was highest among individuals aged 85 and above (41.53; 95 % CI: 41.17 to 41.90, Table 1, Supplementary Fig. 9), followed by those aged 75–84 (13.46; 95 % CI: 13.33 to 13.59, Table 1, Supplementary Fig. 9). Conversely, the lowest death rates were observed in the 15–24 age group (0.06; 95 % CI: 0.06 to 0.0, Table 1, Supplementary Fig. 9) and the 25–34 age group (0.15; 95 % CI: 0.14 to 0.15, Table 1, Supplementary Fig. 9). Similarly, crude death rate for rheumatic MVD was highest among individuals aged 85 and above (14.77; 95 % CI: 14.55 to 14.99, Table 1, Supplementary Fig. 10), followed by those aged 75–84 (7.72; 95 % CI: 7.62 to 7.82, Table 1, Supplementary Fig. 10). Conversely, the lowest death rates were observed in the 5–14 age group (0.01; 95 % CI: 0.01 to 0.01, Table 1, Supplementary Fig. 10) and the 15–24 age group (0.02; 95 % CI: 0.02 to 0.02, Table 1, Supplementary Fig. 10). However, 5–14, 15–24, and 25–34 age groups for rheumatic MVD and 5–14 and 15–24 age groups for nonrheumatic MVD were not analysed due to unreliable data points. Additionally, pairwise comparison was run for age groups 35 years and above. The mortality did not differ significantly across the age group 35–44 of the rheumatic and nonrheumatic subgroups (p = 0.67, Table 2). However, the rates did differ significantly in the age group 45–54 (p = 0.0009, Table 2), 55–64 (p = 0.002, Table 2), 65–74 (p = 0.005, Table 2), 75–84 (p = 0.0008, Table 2) and 85+ (p = 0.0006, Table 2) cohorts.

### 3.6 Trends by state

The AAMRs for nonrheumatic MVD were consistently higher than those for rheumatic MVD across states, with notable differences in magnitude (Fig. 11, Fig. 12***)***. The highest AAMR for nonrheumatic MVD was observed in Vermont (AAMR = 3.39; 95 % CI = 3.10 to 3.67, Fig. 11), approximately 2-fold greater than the highest AAMR for rheumatic MVD, recorded in North Dakota (AAMR = 1.52; 95 % CI = 1.33 to 1.70 Fig. 12). Similarly, the lowest AAMR for nonrheumatic MVD, seen in Georgia (AAMR = 1.14; 95 % CI = 1.09 to 1.19, Fig. 11), was roughly 1.7-fold greater than the lowest AAMR for rheumatic MVD, observed in Louisiana (AAMR = 0.68; 95 % CI = 0.63 to 0.73, Fig. 12).

## 4 Discussion

From 1999 to 2020, mortality trends for rheumatic and nonrheumatic MVD showed distinct patterns across demographics, regions, and age groups. Overall, nonrheumatic MVD had higher mortality rates compared to rheumatic MVD across all categories. Mortality rates for rheumatic MVD initially declined significantly but reversed to show increases after 2017, while nonrheumatic MVD followed a similar pattern with declining rates until 2012, followed by gradual increases. Across sex, race, and geographic subgroups, variations in mortality trends were evident, with the highest AAMRs observed in older adults (85 + ) and non-Hispanic Whites. Urban and rural disparities were noted, with rural areas consistently showing higher AAMRs. State-level trends also highlighted significant variability, with Vermont recording the highest AAMR for nonrheumatic MVD and North Dakota for rheumatic MVD.

The analysis reports a higher AAMR for females with nonrheumatic MVD (1.83) than females with rheumatic MVD (1.09) and their respective male counterparts. Literature confirms the gender-based disparity in epidemiology and morphology [18], attributable to hormonal and anatomical determinants. Fluctuating estrogen levels affect connective tissue remodeling, leading to myxomatous degenerations which are the hallmark of mitral valve prolapse (MVP) [19]. Postmenopausal changes are also linked with increased calcifications of the mitral valve leading to poor outcomes [20]. Under-representation in trials, delayed referrals and limited access to surgical care contribute to a higher mortality risk in females [21]. These factors, along with the tendency of women to experience increased leaflet thickening, anterior or bileaflet mitral valve (MV) prolapse, and higher rates of MV calcification, makes MV repair more challenging and contributes to increased mortality [22]. The study shows a decline in mortality trend for both categories from 1999. This can be attributed to improved diagnostic techniques, advances in medical treatment, and improved surgical techniques for mitral valve repair/replacement [23,24]. The aging population [25] and a rise in comorbidities [26] explain the trend reversal from 2017.

The highest AAMRs for rheumatic MVD and nonrheumatic MVD were observed in NH Whites, consistent with prior epidemiological estimates of RHD-related mortality in the US [27]. It is also consistent with the increased prevalence of advanced cardiovascular risk factors such as smoking [28] and hypertension in this group [29]. Conversely, NH Asians and Hispanics displayed the lowest rheumatic MVD AAMRs, potentially due to genetic predispositions or differing environmental exposures [30]. Notably, the sharp increase in rheumatic MVD mortality among NH African Americans post-2017 (APC: 11.91 %) warrants further investigation into disparities in access to timely intervention and socioeconomic determinants of health. The higher prevalence of homelessness [31,32] and unemployment [33] among African Americans, compared to other racial groups, contributes to their increased likelihood of living in underprivileged neighborhoods [34]. As a result, they often face limited access to health insurance and advanced cardiovascular care. The financial burden associated with RHD management, including long-term care and interventions, can be a significant barrier, especially in low-income populations where access to healthcare is limited.

Nonrheumatic MVD consistently had higher AAMRs compared to rheumatic MVD across all regions. The Midwest showed the highest AAMR for non rheumatic MVD, and the South had the lowest AAMR for rheumatic MVD. However, this is in contrast to findings that were expected, as the South has been identified as a region with a high prevalence of cardiovascular risk factors and major cardiovascular events in comparison to other regions of the US [35]. The South has also been identified as a high obesity spatial regime [36], which makes our findings contrasting to what is expected. Rheumatic MVD in the West had the highest mortality, which may be linked to the region's aging population and the persistence of risk factors such as inadequate treatment for rheumatic fever, especially in underserved communities​. These regional disparities for rheumatic mitral valve degeneration can be closely linked to poor socioeconomic conditions, which elevate the risk of exposure to group A streptococcus by fostering conditions such as overcrowding, inadequate hygiene, and subpar sanitation [[37], [38], [39]].

The study observed higher mortality rates in rural areas compared to urban settings for rheumatic MVD (1.05 vs. 1.00) and nonrheumatic MVD (1.91 vs. 1.78). Rural populations often face systemic barriers, including fewer specialized cardiac centers, delayed diagnosis, and limited emergency care infrastructure [40,41]. Despite emerging telehealth solutions, rural residents remain underserved. Efforts to expand telemedicine programs and mobile heart clinics could alleviate some of these disparities, but structural challenges persist [42,43]. The cost of treating cardiovascular diseases in super-specialty hospitals can be prohibitively high, especially for rural populations often lacking in access to advanced healthcare facilities. Limited availability of specialized care, along with financial barriers, exacerbates the health disparities faced by rural residents, leading to worse outcomes and higher long-term costs [44].

The elderly (aged 85 + ) exhibited the highest mortality rates for both rheumatic MVD and nonrheumatic MVD, reflecting cumulative exposure to risk factors and diminished physiological resilience [45].

Conversely, the youngest cohorts (15–24 years for nonrheumatic MVD and 5–14 years for rheumatic MVD) had the lowest mortality, emphasizing the predominantly age-related nature of these conditions. The disproportionately high mortality among older individuals underscores the need for aggressive early intervention strategies in younger age groups to reduce disease progression and enhance survival outcomes in later life. Poor compliance among diagnosed individuals is a significant factor leading to increased morbidity and mortality in RHD [46].

State-level disparities revealed Vermont's highest nonrheumatic MVD mortality (AAMR: 3.39) and North Dakota's highest rheumatic MVD mortality (AAMR: 1.52). These variations likely reflect differences in population demographics, access to specialized care, and public health infrastructure​.

The lowest AAMRs for nonrheumatic MVD (Nevada: 1.20) and rheumatic MVD (Louisiana: 0.68) may indicate the success of localized health policies and improved care access in these regions. However, state-level resource allocation remains inconsistent, emphasizing the need for federal intervention to standardize care delivery.

The recent increase in mortality rates for both rheumatic and nonrheumatic mitral valve disease (MVD) since 2017 signals a growing concern linked to rising comorbidities like diabetes, hypertension, and obesity. Future research should explore how these risk factors impact MVD progression, especially in aging populations.

Improving access to therapies like transcatheter mitral valve repair (TMVR) is critical, as these treatments remain underused in high-risk groups. Additionally, revising pre-surgical coronary angiography guidelines to prioritize clinical assessments over age alone will help avoid unnecessary procedures and complications.

Addressing health care access disparities, particularly in underserved regions, is essential to reversing the upward trend in mortality. Targeted interventions for early diagnosis and treatment can significantly improve outcomes for MVD patients.

## 5 Limitations

This study benefits from a large dataset spanning two decades, providing a robust overview of rheumatic MVD and nonrheumatic MVD-related mortality trends. However, certain limitations must be noted. Unreported cases and regional variations in healthcare access and quality may affect the findings’ generalizability. The lack of comprehensive data on possible confounders restricts the investigation of complicated variable associations. Inaccuracies in classification of rheumatic and nonrheumatic MVD are also possible, as one may be inaccurately labeled as the other. Our reliance on underlying cause-of-death ICD codes means that cases of RHD complicated by conditions like infective endocarditis (IE) were only captured if RHD was listed as the primary cause. As a result, some deaths attributable to RHD may have been misclassified, limiting a full understanding of the burden of overlapping pathologies. Additionally, the database does not provide information about interventions, and whether these interventions had an effect on mortality. Lastly, the retrospective design restricts the ability to predict future mortality trends that might be mitigated by addressing current disparities.

## 6 Conclusion

The study highlights persistent disparities in mortality trends for rheumatic MVD and nonrheumatic MVD across demographic and geographic groups. An overall rise in mortality trends has been observed in rheumatic and nonrheumatic MVD. The AAMR for nonrheumatic patients was higher than that of rheumatic patients. While early declines in mortality rates reflect healthcare advancements, recent reversals necessitate a renewed focus on addressing systemic inequities and emerging risk factors. Comprehensive public health strategies, improved access to care, and targeted interventions are essential to mitigating these trends and improving survival outcomes.

**Data availability statement**

All data generated or analyzed during this study are included in this published article and its supplementary information files and are freely available on the CDC WONDER database.

## CRediT authorship contribution statement

**Eeman Ahmad:** Writing – review & editing, Writing – original draft, Project administration, Conceptualization. **Shahzaib Ahmed:** Writing – review & editing, Writing – original draft, Visualization, Formal analysis. **Sophia Ahmed:** Writing – original draft, Validation, Investigation. **Hamza Ashraf:** Writing – original draft, Investigation. **Umar Akram:** Writing – original draft, Investigation. **Shoaib Ahmad:** Writing – original draft, Validation, Investigation. **Irfan Ullah:** Writing – review & editing, Validation. **Mohammed Khanji:** Writing – review & editing, Validation. **Wael Awad:** Writing – review & editing, Validation. **Vuyisile Nkomo:** Writing – review & editing, Validation. **Fabrizio Ricci:** Writing – review & editing, Validation. **Matthew Bates:** Writing – review & editing, Validation. **Mohammad Alkhalil:** Writing – review & editing, Validation. **Raheel Ahmed:** Writing – review & editing, Validation. **Anwar A. Chahal:** Writing – review & editing, Validation, Supervision.

## Funding

No funding was received for conducting this research.

## Declaration of competing interest

The authors declare that they have no known competing financial interests or personal relationships that could have appeared to influence the work reported in this paper.
