# Supplementary material for: Comparison of mortality trends in patients with rheumatic mitral valve disease and nonrheumatic mitral valve disease: A retrospective study in US from 1999 to 2020
Source: Int J Cardiol Heart Vasc. 2025 Apr 24;59:101687. doi: 10.1016/j.ijcha.2025.101687 (PMC12060514; doi:10.1016/j.ijcha.2025.101687)

**Supplementary Material**

**Comparison of Mortality Trends in Patients with Rheumatic Mitral Valve Disease and Nonrheumatic Mitral Valve Disease: A Retrospective Study in the United States from 1999 to 2020**

This supplemental material is provided by the authors for a better understanding of their work

**Supplementary Figure 1:** Trends in Rheumatic Mitral Valve Disease-related Mortality stratified by gender in the United States from 1999 to 2020


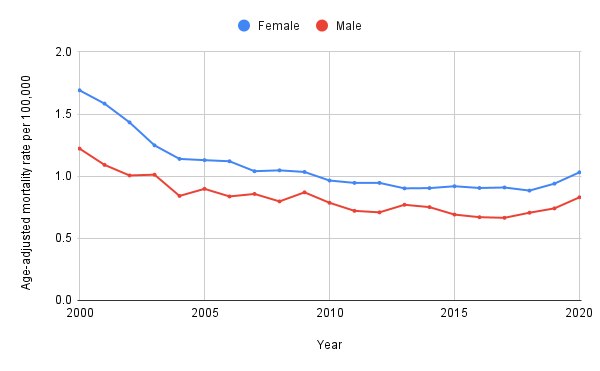


**Supplementary Figure 2:** Trends in Nonrheumatic Mitral Valve Disorder-related Mortality stratified by gender in the United States from 1999 to 2020


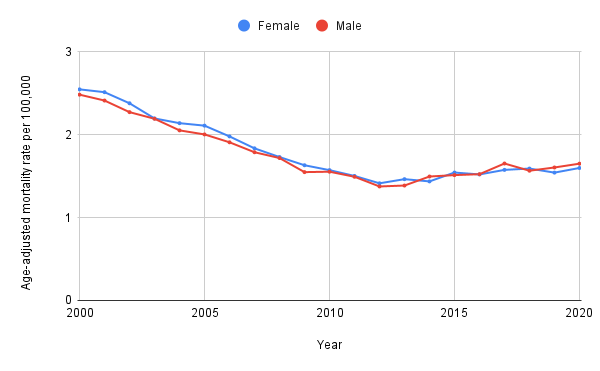


**Supplementary Figure 3:** Trends in Rheumatic Mitral Valve Disease-related Mortality stratified by race in the United States from 1999 to 2020


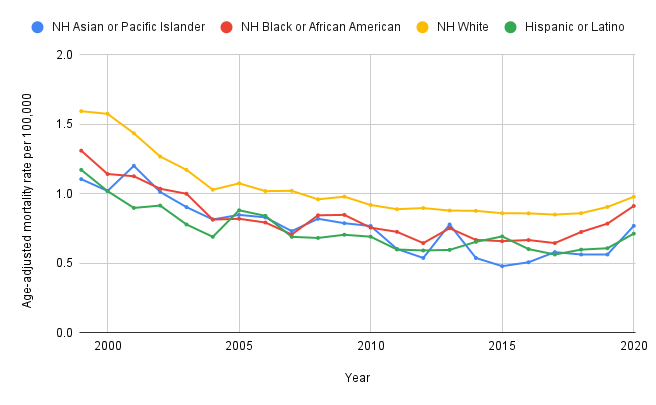


**Supplementary Figure 4:** Trends in Nonrheumatic Mitral Valve Disease-related Mortality stratified by race in the United States from 1999 to 2020


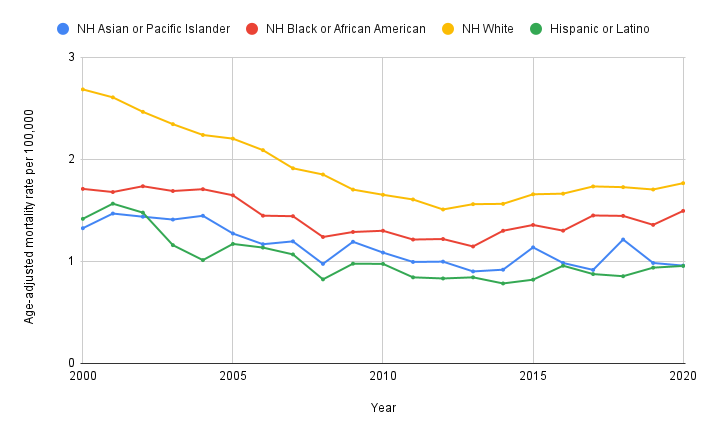


**Supplementary Figure 5:** Trends in Rheumatic Mitral Valve Disease-related Mortality stratified by census region in the United States from 1999 to 2020


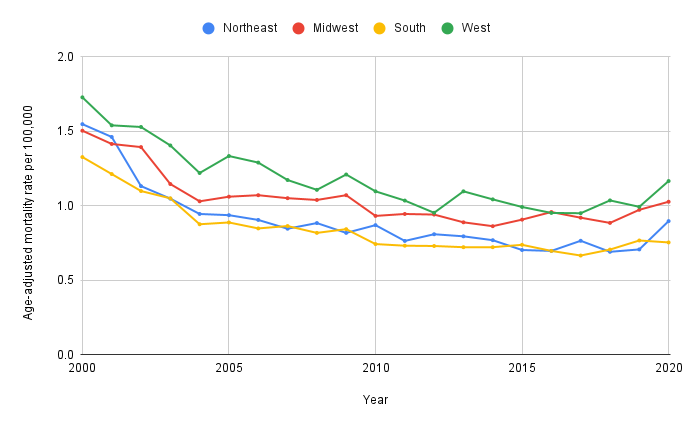


**Supplementary Figure 6:** Trends in Nonrheumatic Mitral Valve Disease-related Mortality stratified by census region in the United States from 1999 to 2020


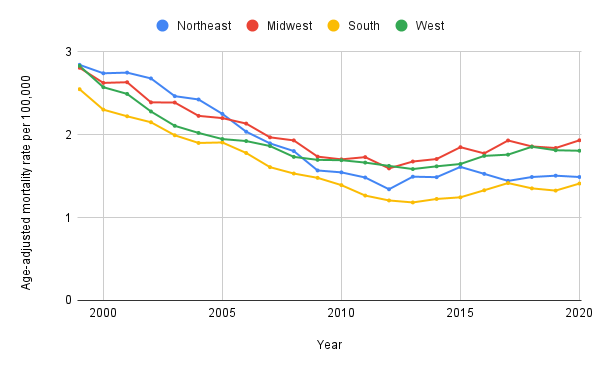


**Supplementary Figure 7:** Trends in Rheumatic Mitral Valve Disease-related Mortality stratified by urbanization in the United States from 1999 to 2020


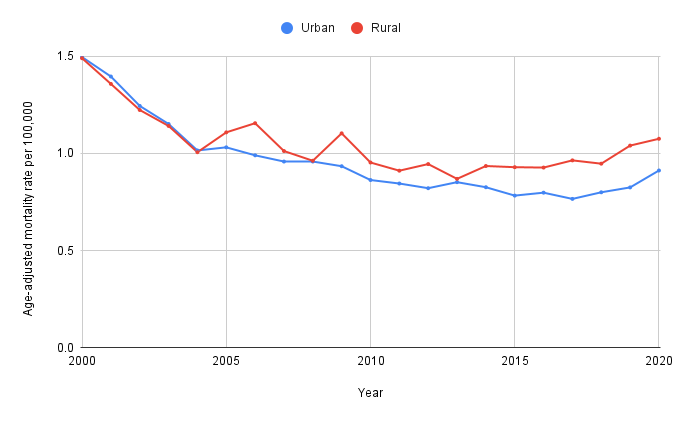


**Supplementary Figure 8:** Trends in Nonrheumatic Mitral Valve Disease-related Mortality stratified by urbanization in the United States from 1999 to 2020


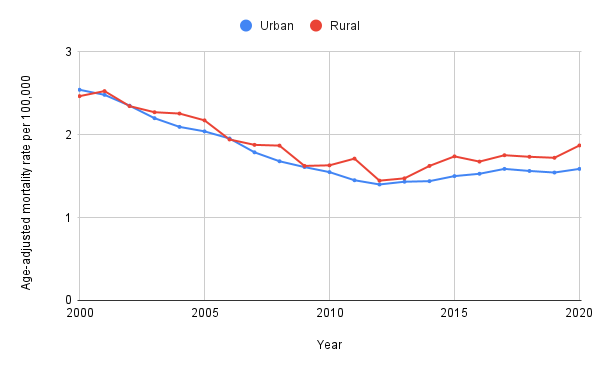


**Supplementary Figure 9:** Trends in Nonrheumatic Mitral Valve Disease-related Mortality stratified by ten-year age groups in the United States from 1999 to 2020


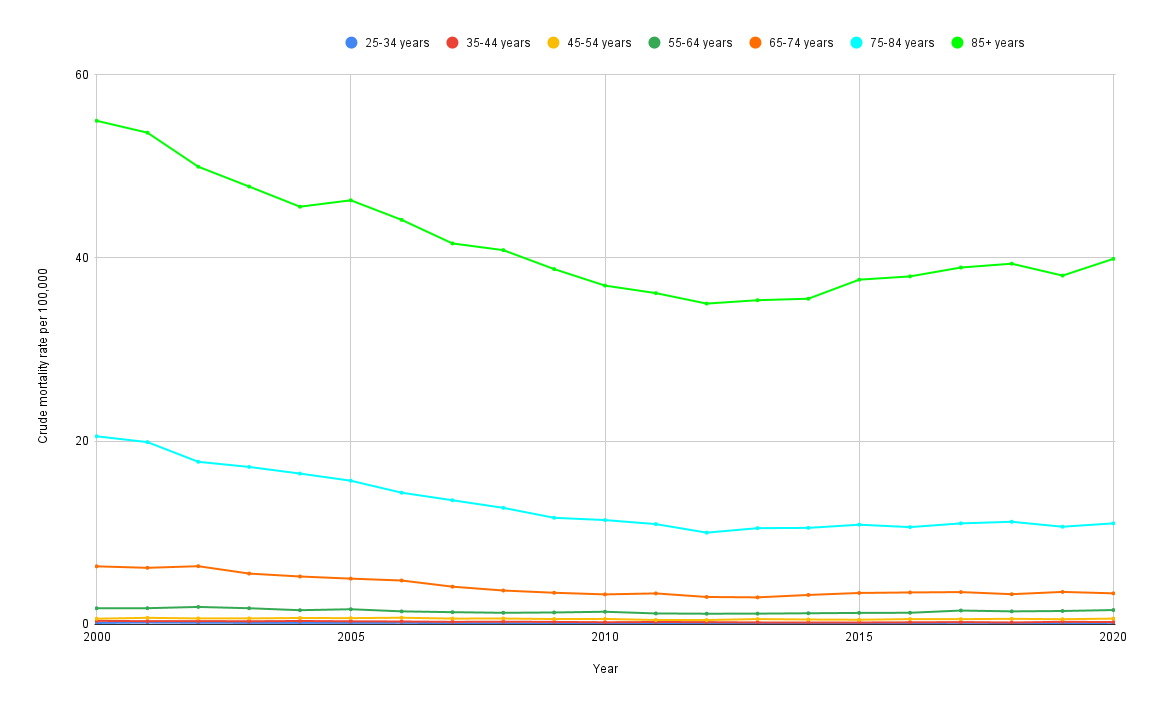


**Supplementary Figure 10:** Trends in Rheumatic Mitral Valve Disease-related Mortality stratified by ten-year age groups in the United States from 1999 to 2020


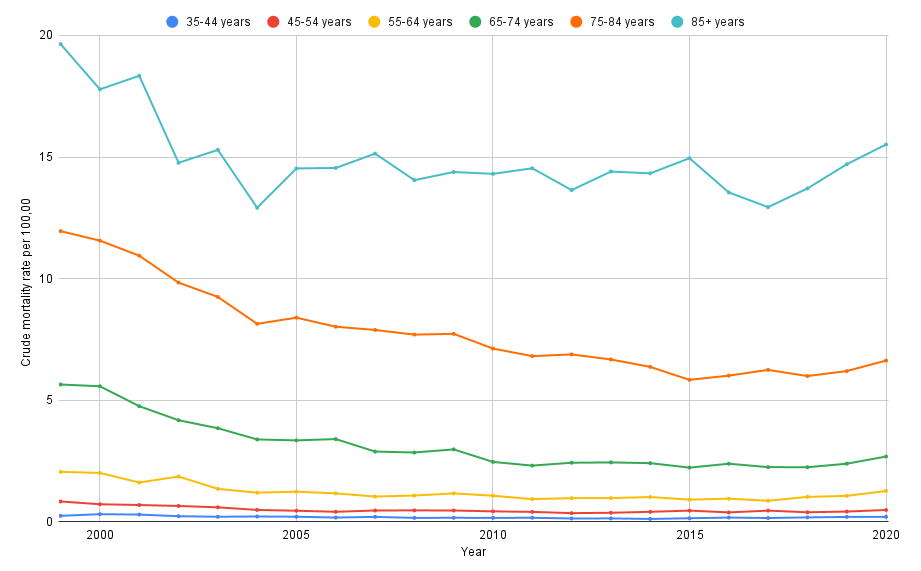


**Supplementary Figure 11:** Trends in Nonrheumatic Mitral Valve Disease-related Mortality stratified by state in the United States from 1999 to 2020


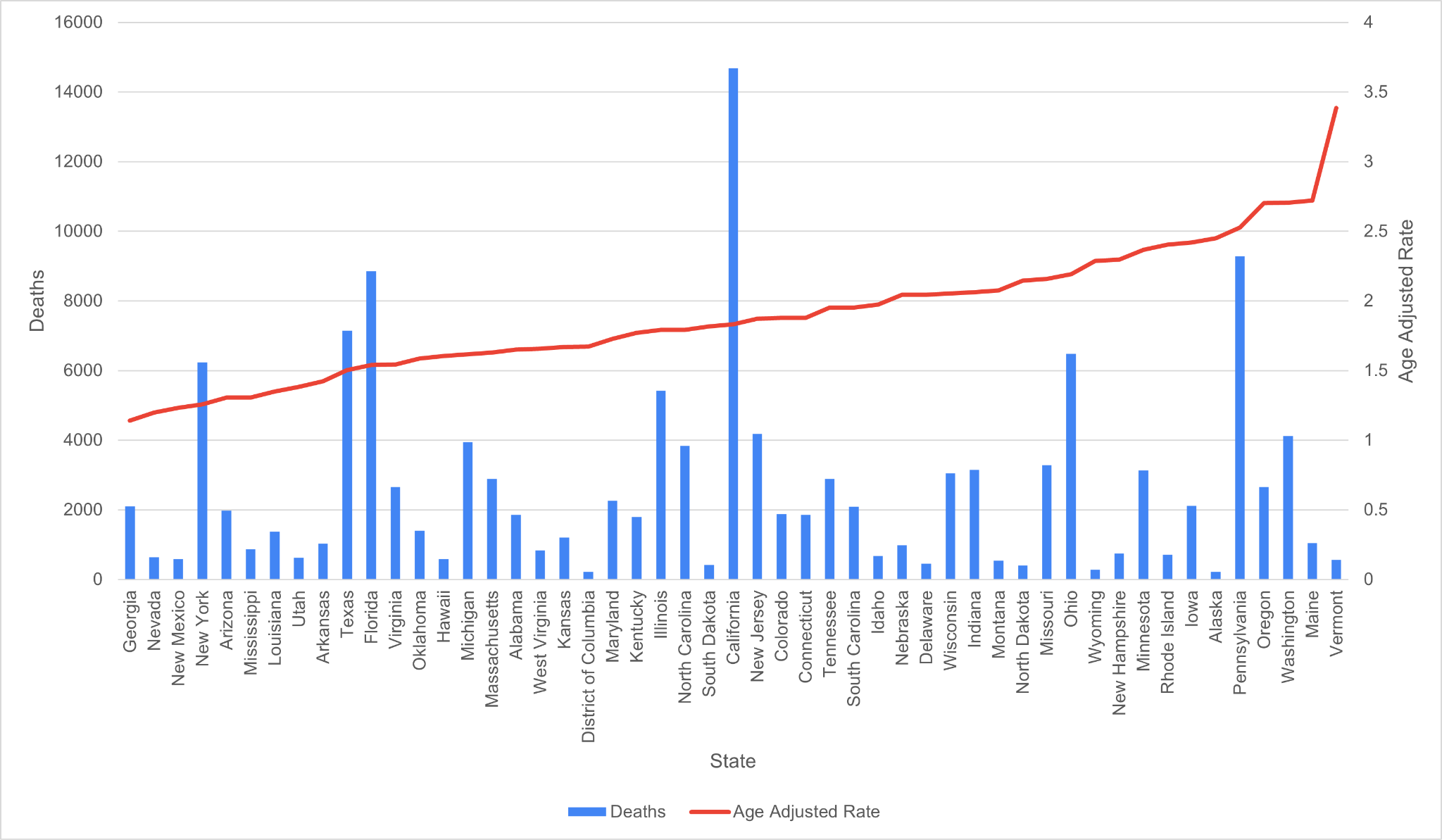

**Supplementary Figure 12:** Trends in Rheumatic Mitral Valve Disease-related Mortality stratified by state in the United States from 1999 to 2020


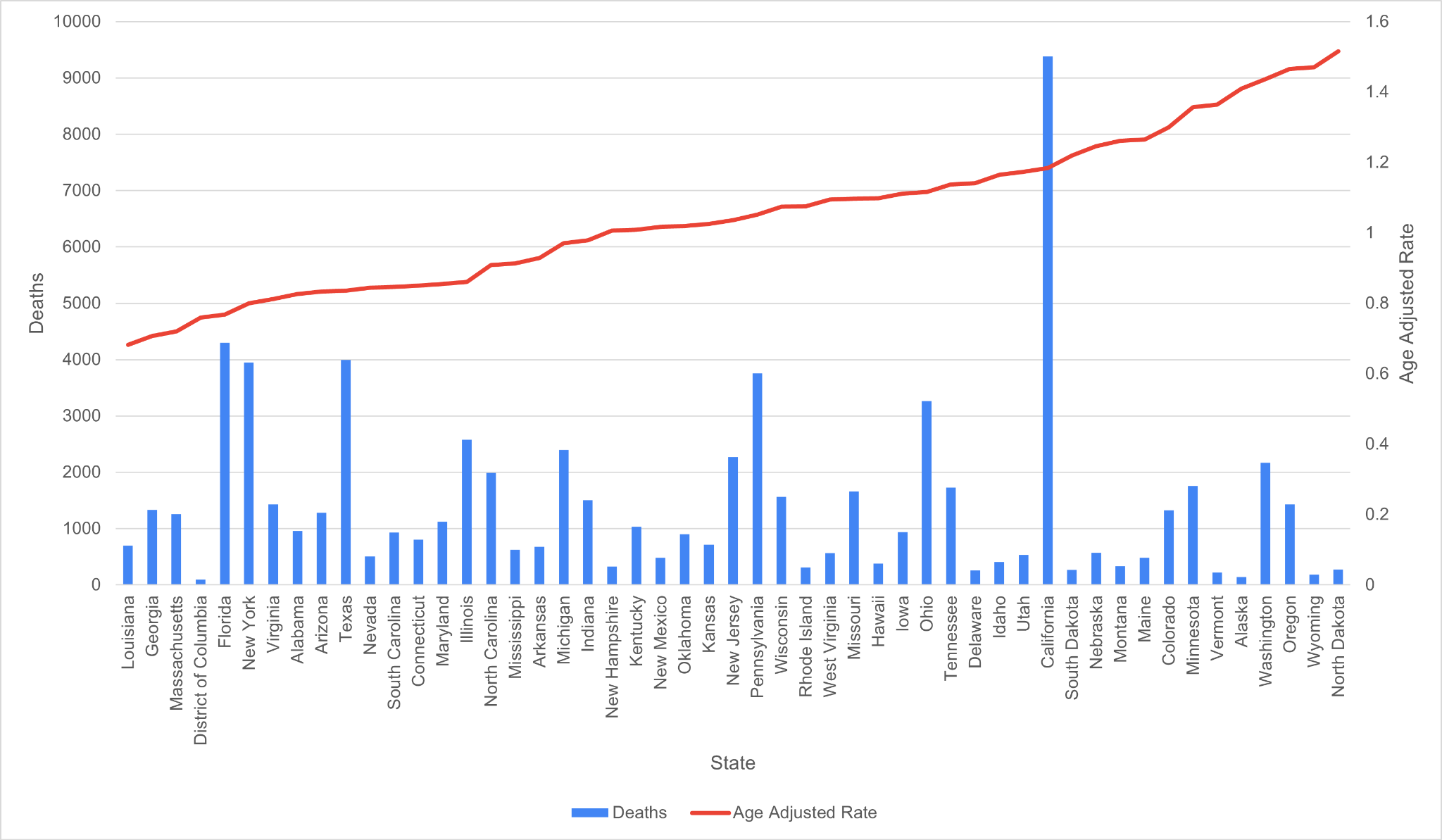

Supplement: Supplementary Data 1 [file mmc1.docx]
